# Microwave-assisted Solvent-free Synthesis and *in Vitro* Antibacterial Screening of Quinoxalines and Pyrido[2, 3b]pyrazines

**DOI:** 10.3390/molecules17055164

**Published:** 2012-05-04

**Authors:** J. Jesús Morales-Castellanos, Karina Ramírez-Hernández, Nancy S. Gómez-Flores, Oscar R. Rodas-Suárez, Javier Peralta-Cruz

**Affiliations:** 1 Department of Organic Chemistry, Escuela Nacional de Ciencias Biológicas del Instituto Politécnico Nacional, Prol. Carpio y Plan de Ayala, México 11340 D. F., Mexico;Email: jesus_qorg@hotmail.com (J.J.M.-C.); chemycal_girl@hotmail.com (K.R.-H.); gomeznancy11@hotmail.com (N.S.G.-F.); 2 Department of Microbiology, Escuela Nacional de Ciencias Biológicas del Instituto Politécnico Nacional, Prol. Carpio y Plan de Ayala, México 11340 D. F., Mexico; Email: rodas07@gmail.com

**Keywords:** 6-substituted quinoxalines, pyrido[2,3*b*] pyrazines, antimicrobial activity

## Abstract

We report herein the microwave assisted synthesis, without solvents and catalysts, of 6-substituted quinoxalines and 7-substituted pyrido[2,3*b*]pyrazines. The compounds were obtained in good yields and short reaction times using the mentioned procedure and two new structures are reported. A complete ^1^H- and ^13^C-NMR assignment was performed using 1D and 2D-NMR. Additionally, an *in vitro* screening was performed on Gram-positive and Gram-negative bacteria using amoxicillin as positive reference. Compounds bearing a pyridyl group tended to have higher antibacterial activity, but the best activity against *Bacillus subtilis* and *Proteus mirabilis* was observed with quinoxaline derivatives.

## 1. Introduction

The most recurrent diseases in North and South American countries are those caused by *Escherichia coli* and *Bacillus subtilis* [1,2]. The antibiotics amoxicillin, norfloxacilin and ciprofloxacin have been the therapeutic agents used to prevent their consequences [3,4,5,6,7], but these drugs have undesired secondary effects and increased chronic toxicity and microbes have developed resistance to most of them [8]. Efforts to prepare antibacterial agents from quinoxaline have resulted in compounds used against a wide variety of disorders, including cancer, diabetes, diabetic retinopathy, rheumatoid arthritis, hemangioma and Kaposi's sarcoma, which are related to vasculogenesis and angiogenesis [9]. The quinoxaline ring moiety is part of the chemical structure of various antibiotics such as echinomycine, levomycine and actinoleutine [10,11] that are known to inhibit growth of Gram-positive bacteria. The mode of action for quinoxalines is based on MAO inhibitory activity, because the docked positions of these compounds reveal interactions with many residues that have an effect on the inhibition of the enzyme [12].

Some quinoxaline derivatives have been synthesized using microwave irradiation [13] but there is no systematic information about their spectroscopic attributes. Synthetic approaches by traditional methodology involves catalysts and solvents [14,15,16,17,18,19], however, recent synthetic methods have focused on techniques involving alternative activation modes. In this sense, microwave-assisted synthesis has shown efficiency and versatility as an easy and convenient methodology for condensation reactions [20,21,22]. It’s known that microwave conditions avoid the polymerization reactions due to the controlled *hot spots* formed during the heating by molecular friction of dipolar molecules [23].

## 2. Results and Discussion

### 2.1. Synthesis and Spectroscopic Analysis

**Scheme 1:**
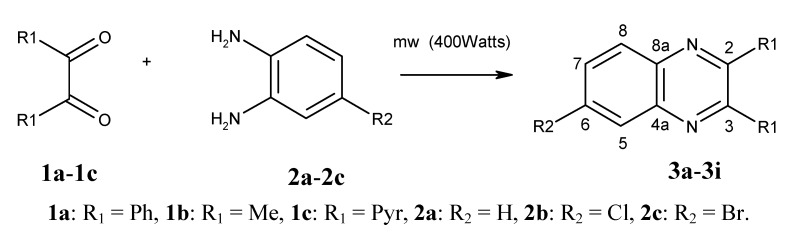
Synthesis of 6-sustituted quinoxalines **3a**–**i**.

**Scheme 2:**
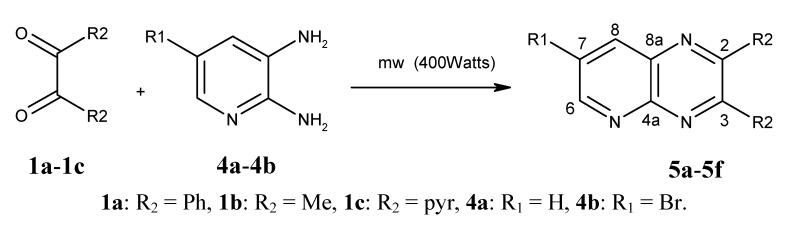
Synthesis of pyrido[2,3*b*]pyrazines **5a**–**f**.

Synthesis of in good yields of quinoxalines **3a**–**i** and pyrido[2,3*b*]pyrazines **5a**–**f** (Schemes 1 and 2) was performed after short reaction times under microwave irradiation. Reactions that involved at least one liquid reagent showed shorter reaction times than those involving two solid reagents (Table 1). Purification of the final crude products with a mixture of hexane-ethyl acetate gave solid pure compounds.

**Table 1:** Reaction times and yields in the synthesis of compounds **3a**–**i** and **5a**–**f**.

Compound	Reaction time (s)	Yield (%)
**3a:** R_1_ = Ph; R_2_ = H	180 ^b^	90
**3b:** R_1_ = Ph; R_2_ = Cl	60 ^b^	85
**3c:** R_1_ = Ph; R_2_ = Br	90 ^b^	88
**3d:** R_1_ = Me; R_2_ = H	60 ^a^	87
**3e:** R_1_ = Me; R_2_ = Cl	30 ^a^	85
**3f:** R_1_ = Me; R_2_ = Br	40 ^a^	84
**3g:** R_1_ = Pyr; R_2_ = H	180 ^b^	84
**3h:** R_1_ = Pyr; R_2_ = Cl	60 ^b^	86
**3i:** R_1_ = Pyr; R_2_ = Br	120 ^b^	80
**5a:** R_1_ = H; R_2_ = Ph	180 ^b^	70
**5b:** R_1_ = H; R_2_ = Me	180 ^b^	60
**5c:** R_1_ = H; R_2_ = Pyr	300 ^b^	80
**5d:** R_1_ = Br; R_2_ = Ph	60 ^b^	85
**5e:** R_1_ = Br; R_2_ = Me	150 ^b^	88
**5f:** R_1_ = Br; R_2_ = pyr	300 ^b^	75

^a^ 2,3-Butanedione is a liquid reagent, diaminopyridines are solid reagents; ^b^ Diaminopyridines and 2,3-dicarbonyl compounds are solid reagents.

Infrared spectra for quinoxalines **3a**–**i** and pyrido[2,3*b*]pyrazines **5a**–**6** showed the characteristic C=N stretching band centered at 1,600 cm^−1^. A weak absorption at 3,050 cm^−1^ was attributed to the =C-H stretching of the aromatic ring. Compounds bearing alkyl groups exhibited a signal at 3,050 cm^−1^ due to C-H stretching absorptions. Bromine derivatives **3c**, **3f**, **3i**, **5d**, **5e** and **5f** showed the typical absorption for C=C-Br stretching at 700 cm^−1^. The ^1^H-NMR analysis of the three coupled aromatic protons H-5, H-7 and H-8 in quinoxalines **3b**, **3c**, **3e**, **3f**, **3h** **and** **3i** were identified as an *AMX* system, showing a double signal for proton H-5 (*J* = 6 Hz), a double of double signal for proton H-7 (*J* = 9 Hz, *J* = 6 Hz) and a double signal for H-8 (*J* = 9 Hz). ^13^C-NMR showed the quinoxaline quaternary carbons C-2 and C-3 of compounds **3a**–**i** ranging from 149–155 ppm.

Quinoxaline carbons of **4a** and **8a** were observed at 135–142 ppm. 6-Substituted quinoxalines **3b**, **3**c, **3e**, **3f**, **3h** and **3i** showed quaternary halogenated carbons around 120 and 132 ppm that correlate with the aromatic protons H-5, H-7 and H-8 in HMBC. Non-planar conformation of vicinal 2–3 biphenyls and 2–3 bipyridyls in compounds **3h**, **3i**, **5a**, **5c**, **5d** and **5f** produced atropoisomeric structures that displayed two ^13^C signals for C-1′, C-2′, C-3′, C-4′ and C-5′ carbons.

**Figure 1:**
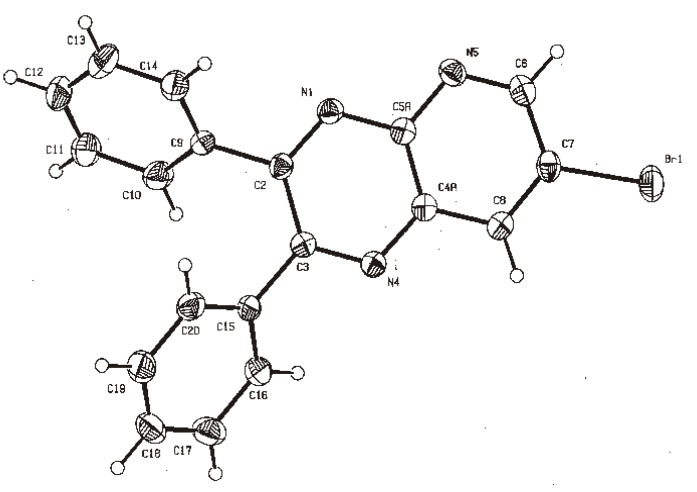
X-Ray diffraction structure of pyrido[2,3*b*] pyrazine **5d**.

X-ray diffraction of compound **5d** (Figure 1) shows the orthogonal conformation of both aromatic benzene rings located on carbons 2 and 3, which is one of the causes for the non-magnetic equivalence of benzene carbons. The same behavior was observed for compounds **5a**, **5c** and **5f**. The ^1^H and ^13^C assignments were made by means of 1D spectra (^13^C, APT and DEPT) and confirmed by 2D spectral analysis (HSQC, HMBC and NOESY). The ^1^H and ^13^C-NMR attributes for these molecules are not reported in current literature; compounds **3i** and **5f** are new molecules.

### 2.2. Antimicrobial Test

Moderate antimicrobial activity (60–100% of amoxicillin) against some Gram-negative and Gram-positive bacteria was observed for quinoxalines **3f**, **3g**, **3h** and **3i** which have a pyridine group in their structure (Table 2). This level of activity could be a consequence of the lipophilic character provided by the pyridine groups. Pyrido[2,3*b*]pyrazines **5c**, **5d**, **5e** and **5f** also showed moderate activity, but primarily against Gram-negative bacteria (Table 2). Recent literature have reported the antifungal properties of pyrido[2,3*b*]pyrazines-*N*-oxides and pyrido[2,3*b*]pyrazinolones [24].

**Table 2:** Antimicrobial activity of compounds **3a**–**i** and **5a**–**f** (200 µM).

Zone of inhibition (mm)									
Compound	*E. coli*	*S. aureus*	*S. typhi*	*E. faecalis*	*S.* *typhimurium*	*P. aeruginosa*	*B. subtilis*	*P. mirabillis*	*S. flexneri*
**3a**	14	7	13	10	9	8	15	8	9
**3b**	11	10	12	9	11	9	11	10	12
**3c**	16	10	11	11	10	8	10	7	15
**3d**	14	8	15	9	8	11	8	9	10
**3e**	12	11	15	10	11	10	9	8	13
**3f**	13	10	14	9	18	9	9	18	14
**3g**	12	15	13	14	10	14	11	17	15
**3h**	14	16	17	15	17	10	17	19	16
**3i**	14	16	10	11	8	9	15	16	12
**5a**	13	14	14	16	15	9	13	12	11
**5b**	11	12	16	12	14	7	15	15	15
**5c**	12	8	11	15	15	12	10	11	14
**5d**	14	14	14	11	14	11	14	9	15
**5e**	15	15	15	14	17	17	12	16	12
**5f**	16	11	14	11	11	14	11	15	12
**Amoxicillin**	22	25	19	18	23	21	19	19	25

Large inhibition zones and a broad spectrum of activities were observed for all the tested organisms except *P. aeruginosa*, which was sensitive to only six of the fifteen compounds screened. The small zones of inhibition exhibited by *P. aeruginosa* may be explained in terms of the gross cell wall of the organism that contains porins and efflux pumps, called ABC transporters, which pump out some antibiotics before they are able to act. It may also be due to the protective biofilms formed by this organism [25].

The results in Table 3 shows that the MIC values of quinoxalines and pyrido[2,3*b*]pyrazines varied between 0.025 µM/mL and 0.1 µM/mL for all bacterial strains. These results indicated that compounds **3g**, **3h**, **5e**, **5f** had the highest activity while **5a**, **5b**, **5c** and **5d** competed favorably. The best zone of inhibition was 19 mm by compound 3h against *P. mirabilis*, which was the same activity as amoxicillin.

**Table 3:** Minimum inhibitory concentration of compounds **3a**–**i** and **5a**–**f**.

Minimum Inhibitory Concentration (µM/mL)									
Comp.	*E. coli*	*S. aureus*	*S. typhi*	*E. faecalis*	*S. typhimurium*	*P. aeruginosa*	*B.* *subtilis*	*P. mirabillis*	*S. flexneri*
**3a**	0.05	>0.2	0.05	0.1	>0.2	>0.2	0.05	>0.2	>0.2
**3b**	0.05	0.1	0.05	>0.2	0.1	>0.2	0.1	>0.2	0.1
**3c**	0.05	0.1	0.1	0.1	>0.2	>0.2	>0.2	>0.2	0.05
**3d**	0.05	>0.2	0.05	>0.2	>0.2	0.1	>0.2	>0.2	>0.2
**3e**	0.05	0.1	0.05	0.1	0.1	>0.2	>0.2	>0.2	0.1
**3f**	0.05	0.1	0.05	>0.2	0.025	>0.2	>0.2	0.025	0.05
**3g**	0.05	0.05	0.05	0.05	>0.2	0.05	0.1	0.025	0.05
**3h**	0.05	0.05	0.025	0.05	0.025	>0.2	0.025	0.025	0.05
**3i**	0.05	0.05	0.1	0.1	>0.2	>0.2	0.1	0.05	0.1
**5a**	0.05	0.05	0.05	0.025	0.05	>0.2	0.1	0.1	0.1
**5b**	0.05	0.1	0.025	0.1	0.05	>0.2	0.1	0.05	0.05
**5c**	0.05	>0.2	0.1	0.05	0.05	0.1	>0.2	>0.2	0.05
**5d**	0.05	0.05	0.05	0.1	0.05	0.1	0.1	>0.2	0.05
**5e**	0.05	0.05	0.05	0.05	0.025	0.025	0.1	0.05	0.1
**5f**	0.05	0.05	0.05	0.1	0.1	0.1	>0.2	0.05	0.1

## 3. Experimental

### 3.1. General

Purity of the compounds was checked by gas chromatography on a Finnigan Trace GC Ultra Polaris Q Chromatograph. Infrared spectra were recorded in CH_2_Cl_2_ solution with a Perkin-Elmer FT-IR 2000 spectrometer. Samples for ^1^H- and ^13^C-NMR were dissolved in CDCl_3_ and spectra recorded on a Varian VNMR System 500 and in a Varian Mercury 300 with TMS as internal standard. Chemical shifts were recorded as δ (ppm). High resolution mass spectra were obtained in a Jeol GCMate II TSS 2000 by electronic impact at 70 eV.

### 3.2. Synthesis

A mixture of the appropriate α-diketone **1a**–**c** (1.1 mmol) and 4-substituted *o*-phenylenediamine **2a**–**c** or 5-substituted 2–3 diaminopyridines **4a**–**b** (1.0 mmol) were mixed in a glass microwave reaction tube. The mixture was placed inside the microwave oven and irradiated at 400 Watts for short periods of time (see Table 1). The reaction was monitored every minute by thin layer chromatography (aluminum silica gel plates F_254_, hexane-ethyl acetate 7:3), and developed with UV light until the reaction was completed. The final time was taken when only traces of *o*-phenylendiamine or 2–3 diaminopyridine were observed by TLC. No solvents or catalysts were used in the synthesis, and any water formed in the reaction was quickly eliminated by evaporation due to the high microwave temperature, eliminating the need to use dehydrating agents. Quinoxalines **3a**–**i** and pyrido[2,3*b*]pyrazines **5a**–**f** were obtained as amorphous crystals. Subsequent crystallization from 7:3 ethyl acetate-hexane gave crystalline or semisolid products in yields ranging from 60–90%. All compound structures were identified from their spectral and analytical data.

### 3.3. Chemical Data and Analysis


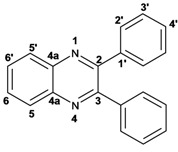


*2,3-Diphenylquinoxaline* (**3a**): mp: 128–129 °C; I.R. (CH_2_Cl_2_): 3058, 1345, 697 cm^−1^; ^1^H-NMR: 8.18 (1H, d, *J* = 5.5 Hz, H_5′_), 8.17 (1H, d, *J* = 3.0 Hz, H_5_), 7.77 (1H, d, *J* = 3.0 Hz, H_6_), 7.76 (1H, d, *J* = 5.5 Hz, H_6′_), 7.51 (4H, m, H_2′_), 7.34 (6H, m, H_3′_, H_4′_); ^13^C-NMR: 155.43 (C_2_, C_3_), 141.20 (C_4a_), 139.05 (C_1′_), 129.90 (C_5_, C_5′_), 129.80 (C_2′_), 129.17 (C_4′_), 128.76 (C_6_, C_6′_), 128.22 (C_3′_); HR MS (*m/z*) 282.1157 (calc.), 282.1156 (exp.).


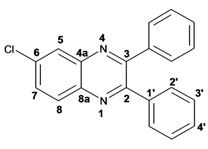


*6-Chloro-2,3-diphenylquinoxaline* (**3b**): mp: 117–119 °C; I.R. (CH_2_Cl_2_): 3060, 1467, 1342, 697 cm^−1^; ^1^H-NMR: 8.15 (1H, d, *J* = 2.5 Hz, H_5_), 8.08 (1H, d, *J* = 9.0 Hz, H_8_), 7.68 (1H, dd, *J* = 2.5, 9.0 Hz, H_7_), 7.50 (4H, d, *J* = 9.0 Hz, H_2′_), 7.34 (6H, m, H_3′_, H_4′_); ^13^C-NMR: 154.51 (C_3_), 153.84 (C_2_), 141.70 (C_6_), 139.93 (C_8a_), 138.93, 138.81 (C_1′_), 135.88 (C_4a_), 130.66 (C_7_), 130.20 (C_8_), 130.08, 130.09 (C_2′_), 129.36, 128.30 (C_3′_), 128.58 (C_5_), 128.30 (C_4_); HR MS (*m/z*) 316.0767 (calc.), 316.0767 (exp.).


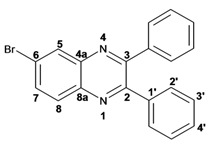


*6-Bromo-2,3-diphenylquinoxaline* (**3c**): mp: 117–119 °C; I.R. (CH_2_Cl_2_): 3059, 1343, 697 cm^−1^; ^1^H-NMR: 8.35 (1H, d, *J* = 1.5 Hz, H_5_), 8.01 (1H, d, *J* = 9.0 Hz, H_8_), 7.80 (1H, dd, *J* = 1.5, 9.0 Hz, H_7_), 7.52 (4H, d, *J* = 7.1 Hz, H_2′_), 7.34 (6H, m, H_3′_, H_4′_); ^13^C-NMR: 154.02 (C_2_), 153.54 (C_3_), 141.58 (C_8a_), 139.77 (C_4a_), 138.58; 138.58 (C_1′_), 132.28 (C_7_), 131.10 (C_5_), 130.35 (C_8_), 129.72; 128.96 (C_2′_), 128.96; 128.89 (C_4′_), 128.16; 128.14 (C_3′_), 123.65 (C_6_); HR MS (*m/z*) 360.0262 (calc.), 360.0262 (exp.).


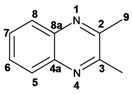


*2,3-Dimethylquinoxaline* (**3d**): mp: 102–104 °C; I.R. (CH_2_Cl_2_): 2995, 1436, 760 cm^−1^; ^1^H-NMR: 7.80 (2H, dd, *J* = 2.0, 6.0 Hz, H_5_, H_8_), 7.47 (2H, dd, *J* = 2.0, 6.0 Hz, H_6_, H_7_), 2.51 (6H, s, H_9_); ^13^C-NMR: 153.48 (C_2_, C_3_), 141.08 (C_4a_, C_8a_), 128.59 (C_5_, C_8_), 128.36 (C_6_, C_7_), 23.19 (C_9_); HR MS (*m/z*) 158.0844 (calc.), 158.0845 (exp.).


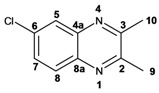


*6-Chloro-2,3-dimethylquinoxaline* (**3e**): mp: 98–100 °C; I.R. (CH_2_Cl_2_): 3022, 1482, 830 cm^−1^; ^1^H-NMR: 7.93 (1H, d, *J* = 2.5 Hz, H_5_), 7.87 (1H, d, *J* = 9.4 Hz, H_8_), 7.57 (1H, dd, *J* = 2.5, 9.4 Hz, H_7_), 2.69 (6H, s, H_9_, H_10_); ^13^C-NMR: 150.03 (C_2_), 149.23 (C_3_), 136.83 (C_8a_), 135 (C_4a_), 129.86 (C_6_), 125.01 (C_5_), 122.81 (C_8_), 18.64 (C_10_), 18.59 (C_9_). HR MS (*m/z*) 192.0453 (calc.), 192.0454 (exp.).


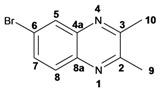


*6-Bromo-2,3-dimethylquinoxaline* (**3f**): mp: 93–94 °C; I.R. (CH_2_Cl_2_): 3019, 1479, 827 cm^−1^; ^1^H-NMR: 7.98 (1H, d, *J* = 2.0 Hz, H_5_), 7.68 (1H, d, *J* = 9.0 Hz, H_8_), 7.57 (1H, dd, *J* = 2.0, 9.0 Hz, H_7_), 2.58 (3H, s, H_9_), 2.56 (3H, s, H_10_); ^13^C-NMR: 154.24 (C_3_), 153.66 (C_2_), 141.31 (C_8a_), 139.43 (C_4a_), 131.86 (C_7_), 130.32 (C_5_), 129.39 (C_8_), 122.00 (C_6_), 22.88 (C_9_, C_10_), HR MS (*m/z*) 235.9949 (calc.), 235.9941 (exp.).


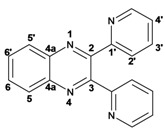


*2,3-Dipyridin-2-yl-quinoxaline* (**3g**). mp: 188–191 °C; I.R. (CH_2_Cl_2_): 2900, 1587, 1351, 744 cm^−1^; ^1^H-NMR: 8.35 (2H, ddd, *J* = 5.0, 2.0, 1.0 Hz, H_5′_), 8.19 (1H, dd, *J* = 8.0, 3.0 Hz, H_5_), 7.94 (2H, ddd, *J* = 8.0, 2.0, 1.0 Hz, H_3′_), 7.80 (2H, m, H_2′_), 7.78 (1H, m, H_6_), 7.20 (2H, ddd, *J* = 10.0, 4.0, 1.0 Hz, H_4′_); ^13^C-NMR: 157.27 (C_1′_), 152.32 (C_2, 3_), 148.46 (C_5′_), 141.07 (C_4a_), 136.59 (C_2′_), 130.43 (C_6_), 129.29 (C_5_), 124.20 (C_3′_), 122.95 (C_4′_); HR MS (*m/z*) 284.1061 (calc.), 284.1062 (exp.).


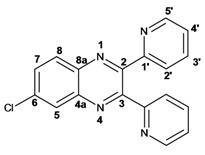


*6-Chloro-2,3-dipyridin-2-yl-quinoxaline* (**3h**): mp: 362–364 °C; I.R. (CHCl_3_): 3059, 1587, 1079, 744 cm^−1^; ^1^H-NMR: 8.32 (2H, m, H_5′_), 8.16 (1H, d, *J* = 2.0 Hz, H_5_), 8.09 (1H, d, *J* = 9.0 Hz, H_8_), 7.91 (2H, ddd, *J* = 8.0, 2.0, 1.0 Hz, H_2′_), 7.72 (1H, dd, *J* = 9.0, 2.0 Hz, H_7_), 7.20 (2H, ddd, *J* = 5.0, 2.0, 1.0 Hz, H_3′_), 7.77 (2H, m, H_4′_); ^13^C-NMR: 156.87, 156.80 (C_1′_), 153.04 (C_3_), 152.40 (C_2_), 148.35, 148.32 (C_5′_), 141.18 (C_4a_), 139.43 (C_8a_) 136.55, 136.52 (C_4′_), 136.09 (C_6_), 131.31 (C_7_), 130.40 (C_8_), 124.09, 124.03 (C_2′_), 122.97, 123.05 (C_3′_); HR MS (*m/z*) 318.0671 (calc.), 318.0672 (exp.).


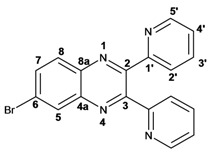


*6-Bromo-2,3-dipyridin-2-yl-quinoxaline* (**3i**): mp: 95–96 °C; I.R. (CH_2_Cl_2_): 3055, 1586, 1077, 745 cm^−1^; ^1^H-NMR: 8.41 (2H, dd, 5,5 Hz, H_5′_), 8.37 (1H, d, *J* = 1.0 Hz), 8.04 (3H, m, H_8, 4′_), 7.89 (3H, m, H_5_, _2′_), 7.34 (2H, dd, *J* = 11, 2 Hz, H_3′_); ^13^C-NMR: 155.81 155.73 (C_1′_), 151.68 (C_3_), 151.11 (C_2_), 147.41, 147.37 (C_5′_), 141.49 (C_4a_) 137.76, 137.72 (C_4′_), 134.29 (C_5_), 131.50 (C_7_), 130.54 (C_8_), 124.81 (C_6_), 124.69, 124.66 (C_2′_), 123.66, 123.60 (C_3′_). HR MS (*m/z*) 362.0169 (calc.), 362.0167 (exp.).


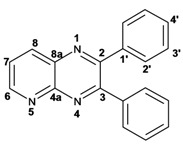


*2,3-Diphenylpyrido[2,3b]pyrazine* (**5a**): mp: 107–109 °C; I.R. (CH_2_Cl_2_): 3058, 1433, 802, 697, cm^−1^; ^1^H-NMR: 9.14 (1H, dd, *J* = 2.0, 5.0 Hz, H_6_), 8.49 (1H, dd, *J* = 2.0, 8.0 Hz, H_8_), 7.68 (1H, dd, *J* = 5.0, 8.0 Hz, H_7_), 7.60 (2H, m, H_2′_), 7.25 (8H, m, H_3′, 4′_); ^13^C-NMR: 156.50 (C_3_), 154.91 (C_2_), 154.07 (C_6_), 150.50 (C_4a_), 138.04 (C_8_), 135.14 (C_8a_), 130.25, 129.81 (C_2′_), 130.12, 129.25 (C_1′_), 129.42, 129.01 (C_4′_), 128.40, 128.14 (C_3′_), 125.21 (C_7_); HR MS (*m/z*) 283.1109 (calc.), 283.1109 (exp.).


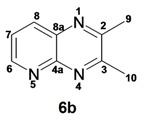


*2,3-Dimethylpyrido[2,3b]pyrazine* (**5b**): mp: 258–260 °C; I.R. (CH_2_Cl_2_): 2994, 1397, 799 cm^−1^; ^1^H-NMR: δ 9.05 (1H, dd, *J* = 2.0, 4.5 Hz, H_6_), 8.33 (1H, dd, *J* = 2.0, 8.5 Hz, H_8_), 7.62 (1H, dd, *J* = 4.0, 8.5 Hz, H_7_), 2.81 (3H, s, H_9_), 2.77 (3H, s, H_10_); ^13^C-NMR 157.22 (C_2_), 154.91 (C_3_), 152.49 (C_6_), 150.12 (C_4a_), 137.20 (C_7_), 135.77 (C_8a_), 124.20 (C_8_), 23.40 (C_9_), 22.94 (C_10_). HR MS (*m/z*) 159.0796 (calc.), 159.0796 (exp.).


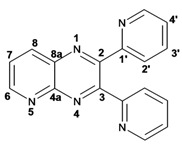


*2,3-Dipyridin-2-yl-pyrido[2,3-b]pyrazine* (**5c**): mp: 418 °C (dec.); I.R. (CH_2_Cl_2_): 3054, 1587, 793 cm^−1^; ^1^H-NMR: δ 9.8 (1H, dd, *J* = 2.0, 4.0 Hz, H_6_), 8.53 (1H, dd, *J* = 2.0, 8.0 Hz, H_7_), 8.35 (1H, d, *J* = 6.0 Hz, H_5′a_), 8.28 (1H, d, *J* = 8.0 Hz, H_4′a_), 8.23 (1H, d, *J* = 6.0 Hz, H_5′b_), 7.94 (1H, d, *J* = 8.0 Hz, H_4’b_), 7.82 (1H, dd, *J* = 2.0, 20.0 Hz H_2′a_), 7.82 (1H, ddd, *J* = 2.0, 15.0, 17.0 Hz, H_2′b_), 7.22 (1H, m, H_3′_); ^13^C-NMR (75 MHz, CDCl_3_): 157.03 (C_1′a_), 156.47 (C_1′b_), 155.17 (C_3_), 154.55 (C_6_), 153.89 (C_2_), 149.62 (C_4a_), 148.55 (C_5′a_), 148.09 (C_5′b_), 138.25 (C_7_), 136.84 (C_2′a_), 136.65 (C_2′b_), 136.23 (C_8a_), 125.65 (C_8_), 124.66 (C_4′a_), 124.08 (C_4′b_), 123.46 (C_3′a_), 123.17 (C_3′b_). HR MS (*m/z*) 285.1012 (calc.), 285.1014 (exp.).


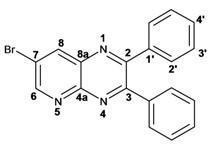


*7-Bromo-2,3-diphenylpyrido[2,3b]pyrazine* (**5d**): mp: 102–104 °C; I.R. (CH_2_Cl_2_): 3055, 1584, 1078, 791 cm^−1^; ^1^H RMN: 9.14 (1H, d, *J* = 2.5 Hz, H_6_), 8.66 (1H, d, *J* = 2.5 Hz, H_8_), 7.61 (1H, d, *J* = 8.0 Hz, H_2′a_), 7.54 (1H, d, *J* = 8.0 Hz H_2′b_), 7.39 (2H, m, H_4′_), 7.36 (1H, d, *J* = 8.0 Hz, H_3′a_), 7.33 (1H, d, *J* = 8.0 Hz, H_3′b_); ^13^C-NMR (75 MHz, CDCl_3_): 156.45 (C_3_), 155.42 (C_2_), 155.04 (C_6_), 148.19 (C_4a_), 139.33 (C_8_), 138.04 (C_1′a_), 137.74 (C_1′b_), 136.35 (C_8a_), 130.13 (C_2′a_), 129.84 (C_2′b_), 129.62 (C_4′a_), 129.54 (C_4′b_), 128.39 (C_3′a_), 128.18 (C_3′b_), 120.86 (C_7_). HR MS (*m/z*) 361.0216 (calc.), 361.0215 (exp). 


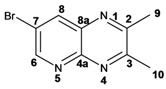


*7-Bromopyrido[2,3b]pyrazine* (**5e**): mp: 320 °C (dec.); I.R. (CH_2_Cl_2_): 3036, 1392, 903 cm^−1^; ^1^H-RMN: 9.04 (1H, d, *J* = 1.0 Hz, H_6_), 8.49 (1H, d, *J* = 1.0 Hz, H_8_), 2.79 (3H, s, H_9_), 2.77 (3H, s, H_10_). ^13^C-NMR: 157.63 (C_3_), 156.05 (C_2_), 153.63 (C_6_), 148.59 (C_2_), 138.79 (C_8_), 136.19 (C_8a_), 119.98 (C_7_), 23.50 (C_9_), 23.05 (C_10_). HR MS (*m/z*) 236.9901 (calc.), 236.9902 (exp.).


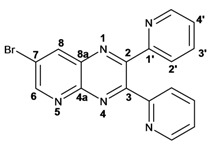


*7-Bromo-2,3-dipyridin-2-yl-pyrido[2,3b]pyrazine* (**5f**): mp: 91–93 °C; I.R. (CH_2_Cl_2_): 2900, 1584, 1390, 744 cm^−1^; ^1^H-NMR: 9.16 (1H, d, *J* = 2.5 Hz, H_6_), 8.69 (1H, d, *J* = 2.5 Hz, H_8_), 8.32 (1H, ddd, *J* = 1.0, 2.0, 5.0 Hz, H_4′a_), 8.24 (2H, ddd, *J* = 1.0, 2.0, 7.0 Hz, H_5′_), 7.95 (1H, ddd, *J* = 1.0, 2.0, 7.0 Hz, H_4′b_), 7.82 (1H, ddd, *J* = 2.0, 15.0, 17.0 Hz, H_2′a_), 7.82 (1H, dd, *J* = 2.0, 15.0 Hz H_2′b_), 7.22 (1H, ddd, *J* = 2.0, 15.0, 17.0 Hz, H_3′a_), 7.22 (1H, ddd, *J* = 1.0, 2.0, 5.0 Hz, H_3′b_); ^13^C-NMR: 156.52 (C_1′a_), 156.2 (C_1′b_), 155.51 (C_6_), 155.30 (C_3_), 154.55 (C_2_), 148.42 (C_5′a_), 148.06 (C_5′b_), 147.91 (C_8a_), 139.46 (C_8_), 136.79 (C_2′a_), 136.63 (C_2′b_), 136.34 (C_4a_), 124.51 (C_4′a_), 124.01 (C_4′b_), 123.5 (C_3′a_), 123.30 (C_3′b_), 121.40 (C_7_). HR MS (*m/z*) 363.0126 (calc.), 363.0120 (exp.).

### 3.4. Antimicrobial Activity

Antimicrobial sensitivity was assessed by the Kirby-Bauer disk diffusion assay [26] using Mueller-Hinton agar plates inoculated with the test organisms seeded in Mueller Hinton medium. Whatman discs (6 mm) were placed on the agar after infusion with a 50, 75, 100, or 200 μM concentration of the test compounds dissolved in DMSO. Amoxicillin was used as a positive reference and DMSO a negative control. The Gram-positive microorganisms tested were *Staphylococcus aureus* (ATCC 6538), *Bacillus subtilis* (ATCC 3465) and *Enterococcus faecalis* (ATCC 29212), and the Gram-negative microorganisms were *Escherichia coli* (ATCC 8739), *Salmonella typhimurium* (ATCC 15674), *Salmonella thypi* (ATCC 6539), *Pseudomonas aeruginosa* (ATCC 9027), *Proteus mirabilis* (ATCC 2756) and *Shigella flexneri* (ATCC 12022). Zones of inhibition were measured by the Bauer protocol [26] after incubating for 24 h at 37 °C.

The minimum inhibitory concentrations (MIC) were evaluated by the micro plate method [27]. 10 µL of each microbial culture, adjusted to 0.5 in the Mc. Farland Nephelometer, were placed in a 96 micro well plate containing 180 µL of Todd-Hewitt Broth medium. Later, 10 µL of the compounds **3a**–**i** and **5a**–**f** at 100, 50, 25 and 12.5 µM concentrations were added to each well. The plate was incubated at 37 °C for 24 h and absorbances measured at 620 nm. Values of MIC_50_ were calculated by following the CLSI (formerly NCCLS) guidelines [27].

## 4. Conclusions

Solvent-free and microwave-assisted synthesis provides an easy, fast and eco-friendly methodology for the preparation of some 6-substituted quinoxalines and pyrido[2,3*b*]pyrazines derivatives from *α*-dicarbonyl derivatives with substituted *o*-phenylenediamines. The antimicrobial Kirby- Bauer test and MIC assay showed moderate activity for some of the prepared quinoxalines **3a**–**i** and analog [2,3*b*] pyridopyrazines **5a**–**f** against a wide group of Gram-positive and Gram-negative bacteria. 

## References

[1] Abu-Ali G.S., Ouellette L.M., Henderson S.T., Whittam T.S., Manning S.D. (2010). Differences in adherence and virulence gene expression between two outbreak strains of enterohaemorrhagic *Escherichia coli* O157:H7. Microbiology.

[2] Logan N.A. (2012). *Bacillus* and relatives in foodborne illness. J. Appl. Microbiol..

[3] Sur D.M., Dutta P., Nair G.B., Bhattacharya S.K. (2000). Severe cholera outbreak following floods in a northern district of West Bengal. Indian J. Med. Res..

[4] Brown J.D., Taylor C.E., Wipf P. (2004). Supplements II Quinoxalines. The Chemistry of Heterocyclic Compounds.

[5] Bhosale R.S., Sarda S.R., Ardhapure S.S., Jadhav W.N., Bhusare S.R., Pawar R.P. (2005). An efficient protocol for the synthesis of quinoxaline derivatives at room temperature using molecular iodine as the catalyst. Tetrahedron Lett..

[6] Minakari M.J., Amir H.D., Shavakhi A., Moghareabed N., Fatahi F. (2010). A randomized controlled trial: Efficacy and safety of azithromycin, ofloxacin, bismuth, and omeprazole compared with amoxicillin, clarithromycin, bismuth, and omeprazole as second-line therapy in patients with *Helicobacter pylori* infection. Helicobacter.

[7] Ozdil B., Kece C., Cosar A., Akkiz H., Sandikci M. (2010). Potential benefits of combined *N*-acetylcysteine and ciprofloxacin therapy in partial biliary obstruction. J. Clin. Pharmacol..

[8] Rezaee M.A., Sheikhalizadeh V., Hasani A. (2011). Detection of integrons among multi-drug resistant (MDR) *Escherichia coli* strains isolated from clinical specimens in Northern West of Iran. Br.J. Microbiol..

[9] Gerspacher M., Furet P., Vangrevelinghe E., Pissot S.C., Gaul C., Holzer P. (2008). Preparation of quinoxalines, particularly heterocyclyl-substituted diarylquinoxalines, as inhibitors of the tyrosine kinase activity of Janus kinases for use in the treatment of immune and proliferative disorders. PCT Int. Appl..

[10] Cheeseman G.W., Cookson R.F., Weissberger A., Taylor E.C. (1979). Condensed pyrazines. The Chemistry of the Heterocyclic Compounds.

[11] Porter A.E., Katritzky A.R., Rees C.W. (1984). An efficient protocol for the synthesis of quinoxaline derivatives at room temperature using molecular iodine as the catalyst. Comprehensive heterocyclic Chemistry.

[12] Sherine N.K., Seham Y.H., Adnan A.B., Abdel M.E., Vratislav L., Adel A. (2010). Synthesis of new series of quinoxaline based MAO-inhibitors and docking studies. Eur. J. Med. Chem..

[13] Jian F.Z., Giu X.G., Li T.A., Yu L., Feng X.Z., Shun J.J. (2008). An efficient synthesis of quinoxalines under catalyst-free and microwave irradiation conditions. Synlett.

[14] Shi D.Q., Dou G.L., Ni S.N., Shi J.W., Li X.Y. (2008). An efficient synthesis of quinoxaline derivatives mediated by stannous chloride. J. Heterocycl. Chem..

[15] Raju B.C., Theja N.D., Kumar J.A. (2009). Efficient and inexpensive synthesis of benzimidazoles and quinoxalines. Synth. Commun..

[16] Liu J.Y., Liu J., Wang J.D., Jiao D.Q., Liu H.W. (2010). Efficient, eco-friendly, and practical process for the synthesis of quinoxalines catalyzed by amberlyst-15 in aqueous media. Synth. Commun..

[17] Krishnakumar B., Swaminathan M.A. (2010). Recyclable and highly effective sulfated TiO2-P25 for the synthesis of quinoxaline and dipyridophenazine derivatives at room temperature. J. Organomet. Chem..

[18] Bandyopadhyay D., Mukherjee S., Rodriguez R.R., Banik B.K. (2010). An effective microwave-induced iodine-catalyzed method for the synthesis of quinoxalines via condensation of 1,2-diamines with 1,2-dicarbonyl compounds. Molecules.

[19] Nandi G.C., Samai S., Kumar R., Singh M.S. (2011). Silica gel-catalyzed efficient synthesis of quinoxaline derivatives under solvent-free conditions. Synth. Commun..

[20] Zhao Z.W., David D., Wolkenberg S.E., Leister W.H. (2004). General microwave-assisted protocols for the expedient synthesis of quinoxalines and heterocyclic pyrazines. Tetrahedron Lett..

[21] Zhou J.F., Gong G.X., Zhi S.J., Duan X.L. (2009). Microwave-assisted catalyst-free and solvent-free method for the synthesis of quinoxalines. Synth. Commun..

[22] Mallouk S., Bougrin K., Laghzizil A., Benhida R. (2010). Microwave-assisted and efficient solvent-free Knoevenagel condensation. A sustainable protocol using porous calcium hydroxyapatite as catalyst. Molecules.

[23] Zhang X.Z., Wang J.X., Bai L. (2011). Microwave-assisted synthesis of quinoxalines in PEG-400. Synth. Commun..

[24] Crowley P.J., Lamberth C., Mueller U., Wendeborn S., Sageot O.A., Williams J., Bartovic A. (2010). Niementowski-type synthesis of pyrido[3,2-e][1,2,4]triazines: Potent aza-analogs of pyrido[2,3-b]pyrazine fungicides. Tetrahedron Lett..

[25] Prescot L.M., Harley J.P., Donald K.A. (2005). Microbiology.

[26] Bauer A.W., Kirby W.M., Sherris J.C., Turek M. (1966). Antibiotic susceptibility by a standardized single disk method. Am. J. Clin. Pathol..

[27] National Committee for Clinical laboratory Standards. 1998. Performance standards for antimicrobial susceptibility testing. 8^th^ international supplement. M100-S8.M2-A6. National Committee for Clinical Laboratory Standards. Wayne, Pa.

